# The complete mitochondrial genomes of *Parabotia kiangsiensis* (Cypriniformes: Botiidae)

**DOI:** 10.1080/23802359.2020.1831990

**Published:** 2020-10-27

**Authors:** Qin Ma, Tonglin Zhang, Lin Chen, Qiongying Tang

**Affiliations:** a Deparment of Biology, Nanchang Normal University, Nanchang, China; b The Key Laboratory of Aquatic Biodiversity and Conservation, Institute of Hydrobiology, Chinese Academy of Sciences, Wuhan, China; c University of Chinese Academy of Sciences, Beijing, China; d College of Life Science, Huzhou University, Huzhou, China

**Keywords:** *Parabotia kiangsiensis*, mitochondrial genome, phylogenetic analysis

## Abstract

*Parabotia kiangsiensis* is an endemic species of genus *Parabotia* in China. In this study, we sequenced the complete mito-genome of *P. kiangsiensis*. The genome is 16,592 base pair (bp) in length, encoding 13 protein-coding genes, 22 tRNA genes, 2 rRNA genes and one non-coding control region (D-loop). The nucleotide composition is A: 30.66%, T: 25.13%, G: 16.3%, and C: 27.92% (AT content:55.79%). The complete mitogenome of *P. kiangsiensis* provides basic data for the genetic diversity conservation of this species.

*Parabotia kiangsiensis* (Cypriniformes, Botiidae, Parabotia), is a small-sized benthic fish endemic to China, mainly distributed in the Poyang basin (Liu and Guo 1986; Guo 2012). It is easy to distinguish *P. kiangsiensis* from other species of genus *Parabotia*, for having two extensive flaky lateral lobes and a free back edge in the lower lip (Liu and Guo 1986). Here, we first determined the complete mitochondrial genome of *P. kiangsiensis* and reconstructed the phylogenetic relationship with other Botiidae species. It may shed light on some genetic background of *P. kiangsiensis*, and could provide basic data for the genetic diversity conservation of this species.

In this study, the specimen of *P. kiangsiensis* were obtained from the Ganjiang River (115°10'E, 27°34'N), Jiangxi, China. Muscles were immediately fixed in 95% ethanol until it was picked out for DNA extraction. Some specimen of *P. kiangsiensis* were preserved in 10% formalin solutions and deposited in Nanchang Normal University (Voucher specimen: NCNU201912001).

Genomic DNA was extracted from muscle by using E.Z.N.A.® Tissue DNA Kit (OMEGA, Beijing, China) following the manufacturer’s instructions. DNA library preparation and 150-bp paired-end sequencing were performed on the Illumina HiSeq platform. After filtering, the mitochondrial data were assembled in NOVOPlasty version 4.0 (Dierckxsens et al. 2017). MITOS was used for annotating the newly sequenced mitogenome. Protein-coding genes (PCGs) and rRNAs were annotated by comparing with the published mitogenomes of the *P. lijiangensis* (Feng and Wang 2020).

The mitogenome of *P. kiangsiensis* was 16,592 bp in length (GenBank with the accession number of MT850132) and contained 13 PCGs (*cyt* b, *ATP6*, *ATP8*, *COX1*-*3*, *ND1*-*6*, *ND4L*), 22 tRNA genes, two rRNA genes (12S and 16S rRNA), and one control region (CR or D-Loop), as other vertebrate mitogenome (Shi et al. 2018; Feng and Wang 2020). Eight tRNA genes (*Gln*, *Ala*, *Asn*, *Cys*, *Tyr*, *Ser*, *Glu* and *Pro*) and NADH dehydrogenase subunit 6 (ND6) are encoded on the light strand (L-strand), the other 29 genes are encoded on the heavy strand (H-strand). The nucleotide composition is A: 30.66%, T: 25.13%, G: 16.3%, and C: 27.92% (AT content:55.79%). Almost all of 13 PCGs for *P. kiangsiensis* share the regular initiation codon ATG except *COI* gene with GTG. There are three different patterns of termination codons: 10 PCGs (terminated with the stop codons TAA or TAG, while three PCGs (*cyt* b, *COX2*, *ND4*) use incomplete stop codon (TA– or T–).

The phylogenetic trees of the genera *Parabotia* and *Leptobotia* were reconstructed based on whole mitogenome dataset. Phylogenetic relationships of *Parabotia* and *Leptobotia* were constructed based on the multiple alignment of 22 mitochondrial genomes within the two genera (Saitoh et al. 2006; Li et al. 2012; Tian et al. 2014; Wan et al. 2014; Tian et al. 2015; Wei et al. 2016a, 2016b) and one outgroup *Botia lohachata* (Yu et al. 2016). ML analysis and NJ analysis were conducted using MEGA7 (Kumar et al. 2016) with 1000 bootstrap replicates. The phylogenetic tree strongly supported the close relationship of *P. banarescui*, *P. lijiangensis,* and *P. Kiangsiensis* (Figure 1). In the tree, *P. banarescui* and *P. kiangsiensis* formed a clade sister to *P. lijiangensis*, which was also congruent with the previous studies (Tang, Xiong, et al. 2005; Slechtová et al. 2006; Tang, Liu, et al. 2006).

**Figure 1.**
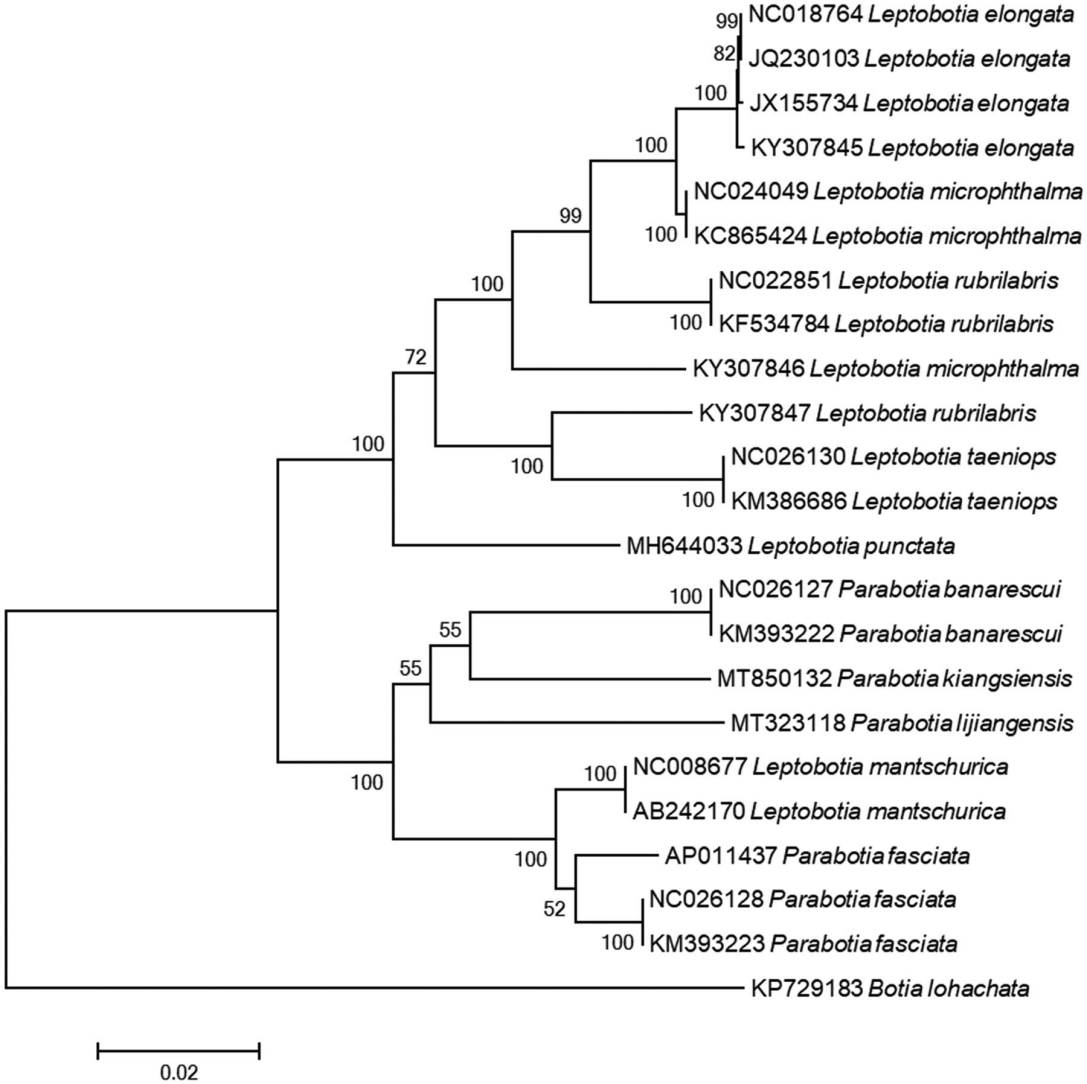
Phylogenetic tree of the genus *Parabotia* and *Leptobotia* using neighbor-joining (NJ) based on whole mitogenome sequences. Values at the nodes correspond to the support values for NJ methods.

## Data Availability

The data that support the findings of this study are openly available in Genbank with the accession codes MT850132 (https://www.ncbi.nlm.nih.gov/nuccore/MT850132).
